# Quackery Defined

**Published:** 1895-09

**Authors:** George F. Shrady


QUACKERY DEFINED.—Quackery is medical practice commercialized, and therefore prostituted. It thrives because the victims are in the majority and are easily reached by lying advertisements. "What is the proportion of sensible people in this crowd?" asked a patent medicine man of a physician. "About one in ten," was the answer. "I take the nine and leave the one to you," said the quack. This represents the majority which help to make the quack rich. The nostrums cost almost nothing; but the capital is used in advertising, in making pictures of the idiots and feeble-minded who imagine themselves cured; in placarding fences; in defacing scenery; in publishing manufactured certificates; in ridiculing scientific medicine; in alarming the credulous; in claiming false discoveries; and in vaunting impossible results. But these are the men who make the money. Medicine to them is the nickel-in-the-slot machine. The diagnosis is ready-made to suit every need, and even otherwise sensible people are being educated into quackery and into the belief that every man can be his own doctor and not have a fool for a patient.—Dr. George F. Shrady in *Forum.*

